# Simplifying screening for *Trypanosoma cruzi* in pregnant persons and their infants

**DOI:** 10.1371/journal.pntd.0011329

**Published:** 2023-05-25

**Authors:** Pierre Buekens, Jackeline Alger, Maria Luisa Cafferata, Eric Dumonteil, Claudia Herrera, Marco Tulio Luque, Yves Carlier

**Affiliations:** 1 Department of Epidemiology, School of Public Health and Tropical Medicine, Tulane University, New Orleans, Louisiana, United States of America; 2 Departamento de Laboratorio Clínico, Hospital Escuela; Instituto de Enfermedades Infecciosas y Parasitología Antonio Vidal; Tegucigalpa, Honduras; 3 Instituto de Efectividad Clínica y Sanitaria, Buenos Aires, Argentina; 4 Department of Tropical Medicine, School of Public Health and Tropical Medicine, Tulane University, New Orleans, Louisiana, United States of America; 5 Servicio de Infectología, Departamento de Pediatría, Hospital Escuela; Servicio de Infectología, Instituto Hondureño de Seguridad Social; Tegucigalpa, Honduras; 6 Laboratoire de Parasitologie, Faculté de Médecine, Université Libre de Bruxelles (ULB), Brussels, Belgium; Hebrew University-Hadassah Medical School, ISRAEL

Mother-to-child transmission is a major source of *Trypanosoma cruzi* infection [1]. The Pan American Health Organization (PAHO) recommends *T*. *cruzi* serological screening of all pregnant persons when part of national policy [2]. The objective of screening pregnant persons is to identify infected infants who could be treated early in life. The World Health Organization (WHO) Technical Group on “Prevention and Control of Congenital Transmission and Case Management of Congenital Infections with *Trypanosoma cruzi*” recommendations are similar to the gold standard approach defined by PAHO [3,4]. The current recommended algorithm for serological testing is that 2 positive tests are required to confirm *T*. *cruzi* seropositivity. If only one of both tests is positive, a third test should be done.

There is a longstanding consensus on the need of a confirmatory test for *T*. *cruzi* infection diagnosis, most likely linked to the fear of overtreating adults with trypanocides, which have many side effects and are of questionable effectiveness in symptomatic adults [5]. In contrast with adults’ treatment, newborns’ and young children’s treatment is effective and has few side effects [6–8]. Requiring 2 positive tests to follow up or treat infants decreases false positives but also increases false negatives. This becomes a significant issue in case of frequent discrepancies between tests, which is often observed in Mexico and Central America [9]. Being falsely classified as negative deprives an infant of being followed up and treated.

**Fig 1:**
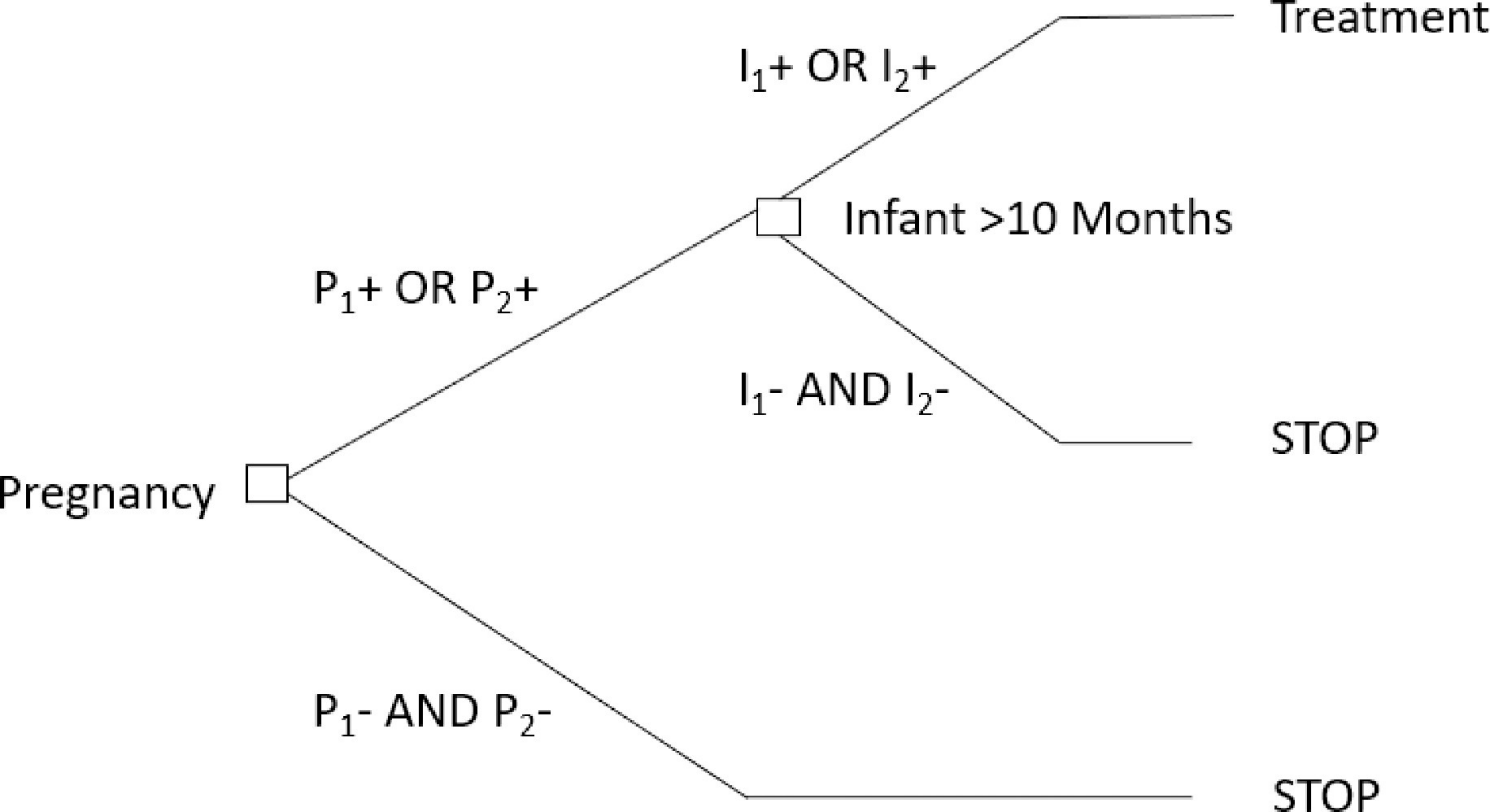
Algorithm including 2 *T*. *cruzi* serological tests during pregnancy (P_1_, P_2_) and in infants of 10 months or more (I_1_, I_2_). P_1_+, P_2_+, I_1_+, I_2_+ refer to positive tests; P_1_−, P_2_−, I_1_−, I_2_− refer to negative tests.

We are proposing a new screening algorithm (Fig 1) that would decrease the number of false negatives, simplify the follow-up, and accelerate access to treatment for children. In this new approach, we continue to recommend 2 serological tests in parallel (including rapid tests), but persons with at least 1 positive *T*. *cruzi* serological test during pregnancy or at delivery (maternal venous blood or cord blood) would be flagged for follow-up. Their infants would be serologically tested after 10 months of age, after the disappearance of maternal antibodies. Again, we recommend 2 serological tests (including rapid tests), but infants would be treated with benznidazole or nifurtimox if at least 1 *T*. *cruzi* serological test is positive. This approach would limit the need for confirmatory tests, simplifying the screening cascade.

The simplified approach we are proposing is inspired by the WHO recommendations for screening for syphilis in pregnancy, based on a single rapid test in low-prevalence settings [10]. We showed in a cluster-randomized controlled trial in Africa that such an algorithm could be effectively implemented [11]. The PAHO strategy to integrate *T*. *cruzi* infection into preventing mother-to-child transmission of HIV and syphilis should encourage us to also simplify our screening approaches for Chagas disease [2,12]. We acknowledge that the positive predictive value of a test decreases with the prevalence, but we feel that the benefit risk balance is in favor of treatment. Pediatric formulations for both benznidazole and nifurtimox have become available in recent years, which facilitates treatment.

We are proposing to rethink the serological strategies to screen for congenital *T*. *cruzi* infection. A simplified algorithm, triggering follow-up and treatment after 1 positive serological test, would allow decreasing false negative rates. It would also simplify the testing cascade. Progress toward elimination of congenital Chagas disease is too slow and access to treatment too limited. We need to find new solutions without further delay.
